# Inequities in hypertension management: observational cross-sectional study in North East London using electronic health records

**DOI:** 10.3399/BJGP.2023.0077

**Published:** 2023-09-19

**Authors:** Stuart Rison, Oliver Redfern, Isabel Dostal, Chris Carvalho, Rohini Mathur, Zahra Raisi-Estabragh, John Robson

**Affiliations:** Clinical Effectiveness Group, Centre for Primary Care, Wolfson Institute of Population Health, Barts and The London School of Medicine and Dentistry, Queen Mary University of London, London; North East London Integrated Care System, Unex Tower, London.; Nuffield Department of Clinical Neurosciences, University of Oxford, Oxford.; Clinical Effectiveness Group, Centre for Primary Care, Wolfson Institute of Population Health, Barts and The London School of Medicine and Dentistry, Queen Mary University of London, London.; Clinical Effectiveness Group, Centre for Primary Care, Wolfson Institute of Population Health, Barts and The London School of Medicine and Dentistry, Queen Mary University of London, London; North East London Integrated Care System, Unex Tower, London.; Clinical Effectiveness Group, Centre for Primary Care, Wolfson Institute of Population Health, Barts and The London School of Medicine and Dentistry, Queen Mary University of London, London.; National Institute for Health and Care Research (NIHR) Academic Clinical Lecturer in Cardiology, Barts Heart Centre, St Bartholomew’s Hospital, Barts Health NHS Trust, London; William Harvey Research Institute, NIHR Barts Biomedical Research Centre, Queen Mary University London, London.; Clinical Effectiveness Group, Centre for Primary Care, Wolfson Institute of Population Health, Barts and The London School of Medicine and Dentistry, Queen Mary University of London, London.

**Keywords:** antihypertensives, blood pressure, cardiovascular diseases, general practice, hypertension, health inequities

## Abstract

**Background:**

Hypertension is a key modifiable risk factor for cardiovascular disease – the leading cause of death in the UK. Good blood pressure (BP) control reduces mortality. However, health inequities may lead to variability in hypertension monitoring and control.

**Aim:**

To investigate health inequities related to ethnicity, sex, age, and socioeconomic status in the monitoring, treatment, and control of BP in a large cohort of adult patients with hypertension.

**Design and setting:**

A cross-sectional cohort study of adults with hypertension registered with general practices in North East London on 1 April 2019.

**Method:**

Multivariable logistic regression was used to estimate associations of demographics and treatment intensity for the following hypertension management indicators: a) BP recording in past 12 months; b) BP on age- adjusted target; and c) BP on age-adjusted target and BP recorded in past 12 months.

**Results:**

In total, 156 296 adults were included. The Black ethnicity group was less likely to have controlled BP than the White ethnicity group (odds ratio [OR] 0.87, 95% [confidence interval] CI = 0.84 to 0.91). The Asian ethnicity group was more likely to have controlled BP (OR 1.28, 95% CI = 1.23 to 1.32). Ethnicity differences in control could not be explained by the likelihood of having a recent BP recording, nor by treatment intensity differences. Older adults (aged ≥50 years) were more likely to have controlled hypertension than younger patients.

**Conclusion:**

Individuals of Black ethnicity and younger people are less likely to have controlled hypertension and may warrant targeted interventions. Possible explanations for these findings are presented but further research is needed about reasons for ethnic differences.

## INTRODUCTION

Cardiovascular disease (CVD) remains the leading cause of death in the UK. In the past decade, the reduction in CVD mortality has stalled and, for some disadvantaged groups, mortality rates have actually increased.^1^^–^3 The importance of effective control of blood pressure in people with hypertension is recognised at local, national, and international level as a major contributor to reduction in CVD-related morbidity and mortality.^4^^–^7

Health inequities in the management of hypertension have been investigated in several regional and national patient cohorts in the UK and these have found the Black ethnicity group to be significantly less likely to have controlled blood pressure than the White ethnicity group and, in some studies, the Asian ethnicity group to have better controlled blood pressure.^8^^–^10 Similar inequities have been described in Europe and the US.^11^^,^^12^

The North East London locations investigated in the current study are among the most ethnically diverse and deprived in the UK.^13^ However, supported by local quality improvement programmes, the control of blood pressure was among the best in England in the national NHS Quality and Outcomes Framework (QOF) reward and incentive programme up until the pandemic.^14^

Regular monitoring of blood pressure and appropriate treatment initiation and escalation are fundamental in effective management of hypertension.^5^ The current study included adults with hypertension and aimed to analyse sociodemographic inequities in recording and control of blood pressure, and inequities in treatment intensity.

## METHOD

A cross-sectional study was carried out in five contiguous North East London clinical commissioning groups (CCGs): City and Hackney; Newham; Redbridge; Tower Hamlets; and Waltham Forest. The study cohort was derived from currently registered patients in general practices that used the EMISWeb electronic health record system (EMIS Health, Leeds). Pseudonymised coded demographic and clinical data were extracted by the study’s data analyst from CCG-level GP electronic health records using the EMISWeb ‘Population Reporting’ tool.

**Table:** How this fits in

Health inequities in the management of long-term conditions are widely recognised. This study identifies ethnic, age, sex, and deprivation inequities in the monitoring, treatment, and control of blood pressure in a large, unselected cohort of adults with hypertension in an ethnically diverse and nationally disadvantaged area of London. It confirms previous findings that blood pressure control in Black ethnic groups with hypertension is worse than in White or Asian ethnic groups. These differences were not related to access to blood pressure recording or treatment intensity. Younger adults (aged <50 years) were less likely to have controlled hypertension than older adults.

The study cohort comprised adults aged ≥18 years, with diagnosed hypertension on the index date: 1 April 2019. The QOF ‘HYP_COD’ reference codeset identified hypertension, excluding individuals with a subsequent ‘hypertension resolved’ code (see Supplementary Box S1 and https://clinicalcodes.rss.mhs.man.ac.uk/medcodes/article/200).^15^ For each cohort individual, the following demographic data were extracted: age in years on index date; sex; home lower layer super output areas (LSOA); and ethnic group code (see Supplementary Box S2).

**Figure 1.**
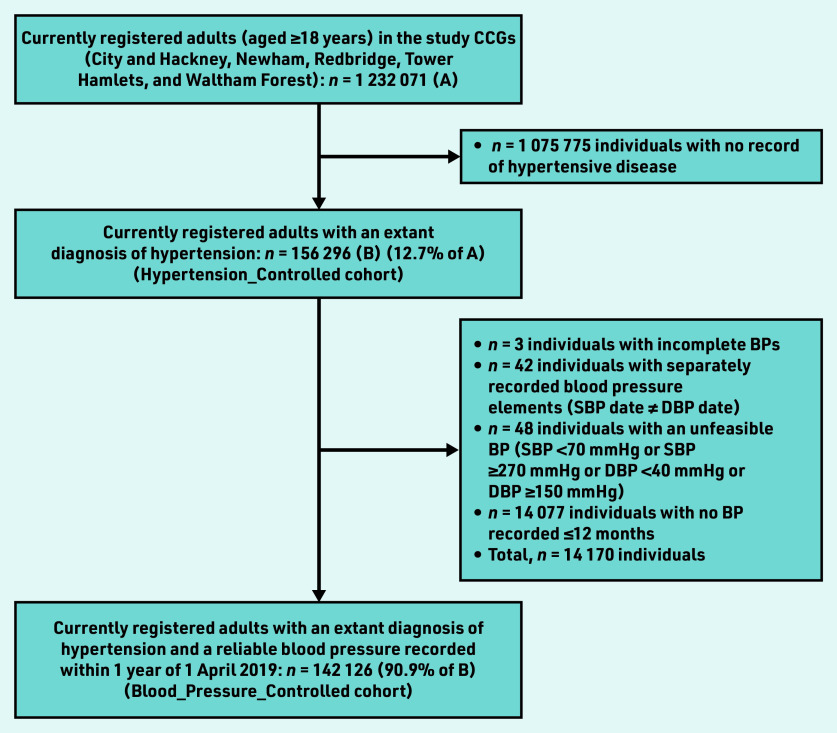
*Study population flowchart. BP = blood pressure. CCG = clinical commissioning group. DBP = diastolic BP. SBP = systolic BP.*

The most recent systolic blood pressure (SBP) and diastolic blood pressure (DBP) values (mmHg) and their entry dates were extracted. Blood pressure recordings made >12 months before the index date were omitted. Blood pressure recordings were also omitted for individuals with unreliable or unfeasible blood pressures, namely: incomplete recording (SBP but no DBP, or vice-versa); separately recorded blood pressures elements (SBP date different from DBP date); and SBP <70 mmHg or SBP ≥270 mmHg or DBP <40 mmHg or DBP ≥150 mmHg (Figure 1).

Medicines prescribed in the 6 months up to and including the index date were considered for eight classes of antihypertensive medication (see Supplementary Box S2):

- angiotensin-converting enzyme inhibitors/angiotensin receptor blockers;
- beta-blockers;
- potassium-sparing diuretics;
- calcium channel blockers;
- thiazide-type and thiazide-like diuretics;
- centrally acting antihypertensives;
- alpha-blockers; and
- loop diuretics.

Only the most recently prescribed antihypertensive in each medication class was considered. Most individuals with hypertension require ≥2 antihypertensive drugs to maintain their blood pressures within an acceptable range.^6^^,^^16^^,^^17^ The number of different classes prescribed were therefore grouped into categoric treatment intensity: 0 (untreated); 1 (possibly undertreated); or ≥2 medications.

Ethnic groups were categorised according to top-level Office for National Statistics 2001 census categories and comprised: White (including British, Irish, and White other); Black and Black British (including Caribbean, African, and other Black background); Asian and Asian British (including Bangladeshi, Pakistani, Indian, and any other Asian background); Mixed ethnicity (including White and Black Caribbean, White and Black African, and White and Asian); and other ethnic groups.^18^ The unknown ethnicity group comprised individuals with no ethnicity code recorded, unclassified codes, and the ‘not stated’ codes.

An individual’s Index of Multiple Deprivation (IMD) was based on their small area indicators (LSOA) and used the 2019 national quintiles from quintile 1 (most deprived) to quintile 5 (least deprived).^19^

Blood pressure was deemed controlled as per NHS QOF indicators HYP003 and HYP007: individuals <80 years of age: SBP ≤140 mmHg and DBP ≤90 mmHg (HYP003); individuals ≥80 years of age, SBP ≤150 mmHg and DBP ≤90 mmHg (HYP007).^15^

### Outcomes

Three binary outcome variables of blood pressure management were considered:

- Blood_Pressure_Recorded was true of individuals with a valid blood pressure recorded within 12 months of the index date;
- Hypertension_Controlled considered the entire cohort (that is, individuals with hypertension) and was true for patients with a blood pressure measure within the QOF age-adjusted target. Individuals without a recorded blood pressure within 12 months of the index date were considered to be above target blood pressure; and
- Blood_Pressure_Controlled considered only individuals with both blood pressure recorded within 12 months of the index date and with blood pressure within the QOF age-adjusted target.

The difference between Hypertension_ Controlled and Blood_Pressure_Controlled is worth clarifying. The former aligns with the NHS’ QOF management of hypertension indicators. QOF takes a ‘worst-case scenario’ approach: individuals on the hypertension register who do not have a blood pressure recorded in the past 12 months are deemed to have uncontrolled hypertension.^15^ However, it is unlikely that all individuals with no blood pressure recorded in the past year actually have uncontrolled blood pressure. By considering only individuals with a blood pressure recorded in the past 12 months, as is the case with this study’s Blood_Pressure_Controlled indicator, a more reliable estimate of overall management of blood pressure can be obtained. Here, the authors used Hypertension_Controlled to simplify comparison of this study’s findings with other audits that treat ‘out of date’ blood pressure recordings as the QOF does, and Blood_Pressure_Controlled as the author’s preferred indicator of blood pressure control (see Discussion).

### Statistics

Data were processed, aggregated, and validated, and descriptive statistics derived using Python (version 3.9.1). Univariate and multiple logistic regression analyses (adjusted for sex, age, ethnicity, IMD quintile, and treatment intensity) were performed using R (version 4.0.5), with subanalyses performed by treatment intensity. Forest plots were generated using the forestplot Python package (version 0.2.0).

## RESULTS

The study cohort was derived from 190 out of 199 practices in the participating five North East London CCGs with a population of approximately 1.23 million adults, of whom 156 296 (12.7%) had hypertension. The nine excluded practices used a different electronic health record system.

The summary characteristics of the study cohort are described in Table 1. There were 14 077 individuals who did not have blood pressure recorded in the 12 months up to and including the index date, and a further 93 blood pressure recordings were excluded as unreliable or not feasible (Figure 1). Therefore, among the whole cohort, 90.9% (*n* = 142 126/156 296) had a valid blood pressure recorded in the 12 months before and including the index date (Blood_Pressure_Recorded).

Using the entire cohort (all missing blood pressure values deemed uncontrolled), that is Hypertension_Controlled, 73.4% (*n* = 114 653/156 296) had controlled blood pressure to age-adjusted targets. Of those with recorded blood pressures, 80.7% (*n* = 114 653/142 126) had controlled blood pressure as per age-adjusted targets (Blood_Pressure_Controlled) (data not shown).

**Table 1.** Characteristics of patients included in the study cohort (*N* = 156 296 adult patients with hypertension)

**Characteristic**	***n* (%)^a^**
**Individual patients**	156 296

**Age, years, mean (SD)**	61.2 (13.9)

**Systolic blood pressure, mmHg, mean (SD)**	133.4 (14.0)

**Diastolic blood pressure, mmHg, mean (SD)**	78.4 (10.1)

**Sex**	
Female	79 940 (51.1)^b^
Male	76 356 (48.9)

**Ethnicity**	
White	59 897 (38.3)^b^
Asian or Asian British	46 856 (30.0)
Black or Black British	33 731 (21.6)
Unknown	6322 (4.0)
*No ethnicity code recorded*	4279 (2.7)
*Unclassified code*	1089 (0.7)
*‘Not stated’ code*	954 (0.6)
Other ethnic groups	6256 (4.0)
Mixed	3234 (2.1)

**Age distribution, years**	
18–29	894 (0.6)
30–39	6063 (3.9)
40–49	19 325 (12.4)
50–59	37 970 (24.3)
60–69	39 854 (25.5)^b^
70–79	30 195 (19.3)
80–89	18 264 (11.7)
90–120	3731 (2.4)

**IMD quintile**	
Q1 (most deprived)	41 364 (26.5)
Q2	72 009 (46.1)^b^
Q3	26 242 (16.8)
Q4	11 801 (7.6)
Q5 (least deprived)	4820 (3.1)
Q0 (unknown)	60 (<0.1)

**Number of antihypertensive medications^c^**	
0	20 365 (13.0)
1	53 492 (34.2)^b^
2	48 635 (31.1)
3	23 373 (15.0)
4	8120 (5.2)
≥5	2311 (1.5)

a *Unless otherwise stated.*

b *Indicates the largest top- level category in variables with multiple categories.*

c *The sum (%) for categories 2 to ≥5 combined was 82 439 (52.7%). IMD = Index of Multiple Deprivation. Q = quintile.*

In total, 96.0% (*n* = 149 974/156 296) of the entire cohort had an ethnicity code assignable to one of the five known ethnicity groups considered in this study, and only 2.7% (*n* = 4279/156 296) of cohort individuals had no ethnicity code recorded (Table 1).

### Blood pressure recording and ethnicity

Blood_Pressure_Recorded and ethnicity were modelled using unadjusted and adjusted logistic regression in relation to the White ethnicity group (Table 2). In the univariate model, the Asian ethnicity group was 60% more likely to have a recent blood pressure recorded than the White ethnicity group (odds ratio [OR] 1.60, 95% confidence interval [CI] = 1.53 to 1.68, *P*<0.001); all other ethnicity groups (except for the unknown ethnicity group) were all within 10% of the reference White ethnic group (Table 2 and Supplementary Figure S1a).

**Table 2.** Binomial logistic regression modelling of the impact of ethnicity on blood pressure control as assessed by the Blood_ Pressure_Recorded, Hypertension_Controlled, and Blood_Pressure_ Controlled indicators

**Ethnicity**	**Logistic regression model**			

	**Unadjusted^a^**		**Adjusted^b^**	

	**OR (95% CI)**	***P*-value**	**OR (95% CI)**	***P*-value**
**Blood_Pressure_Recorded^c^**				
Asian or Asian British	1.60 (1.53 to 1.68)	<0.001	1.62 (1.55 to 1.71)	<0.001
Other ethnic group	1.10 (1.01 to 1.20)	0.04	1.14 (1.03 to 1.25)	0.008
Black or Black British	1.08 (1.03 to 1.13)	<0.001	1.09 (1.03 to 1.14)	0.001
White	Ref	Ref	Ref	N/A
Mixed	0.96 (0.85 to 1.07)	0.43	0.99 (0.87 to 1.12)	0.85
Unknown	0.56 (0.52 to 0.60)	<0.001	0.69 (0.64 to 0.74)	<0.001

**Hypertension_Controlled^d^**				
Asian or Asian British	1.26 (1.22 to 1.30)	<0.001	1.39 (1.35 to 1.43)	<0.001
Other ethnic groups	1.17 (1.10 to 1.24)	<0.001	1.26 (1.18 to 1.34)	<0.001
White	Ref	Ref	Ref	N/A
Black or Black British	0.90 (0.87 to 0.93)	<0.001	0.92 (0.89 to 0.95)	<0.001
Mixed	0.85 (0.79 to 0.92)	<0.001	0.90 (0.83 to 0.98)	0.011
Unknown	0.63 (0.60 to 0.67)	<0.001	0.75 (0.71 to 0.79)	<0.001

**Blood_Pressure_Controlled^e^**				
Other ethnic group	1.34 (1.25 to 1.44)	<0.001	1.29 (1.20 to 1.39)	<0.001
Asian or Asian British	1.23 (1.20 to 1.27)	<0.001	1.28 (1.23 to 1.32)	<0.001
White	Ref	Ref	Ref	N/A
Black or Black British	0.92 (0.89 to 0.95)	<0.001	0.87 (0.84 to 0.91)	<0.001
Mixed	0.90 (0.83 to 0.99)	0.023	0.87 (0.80 to 0.96)	0.003
Unknown	0.78 (0.73 to 0.83)	<0.001	0.82 (0.76 to 0.87)	<0.001

a *Unadusted: indicator ∼ ethnicity.*

b *Adjusted: indicator ∼ ethnicity, sex, age group, IMD quintile, and treatment intensity.*

c *Likelihood of having blood pressure recorded up to 1 year before the index date.*

d *Likelihood of blood pressure meeting age-adjusted blood pressure target, whole cohort.*

e *Likelihood of blood pressure meeting age-adjusted blood pressure target in patients with a blood pressure recorded up to 1 year before the index date. N/A = not applicable. OR = odds ratio.*

**Figure 2.**
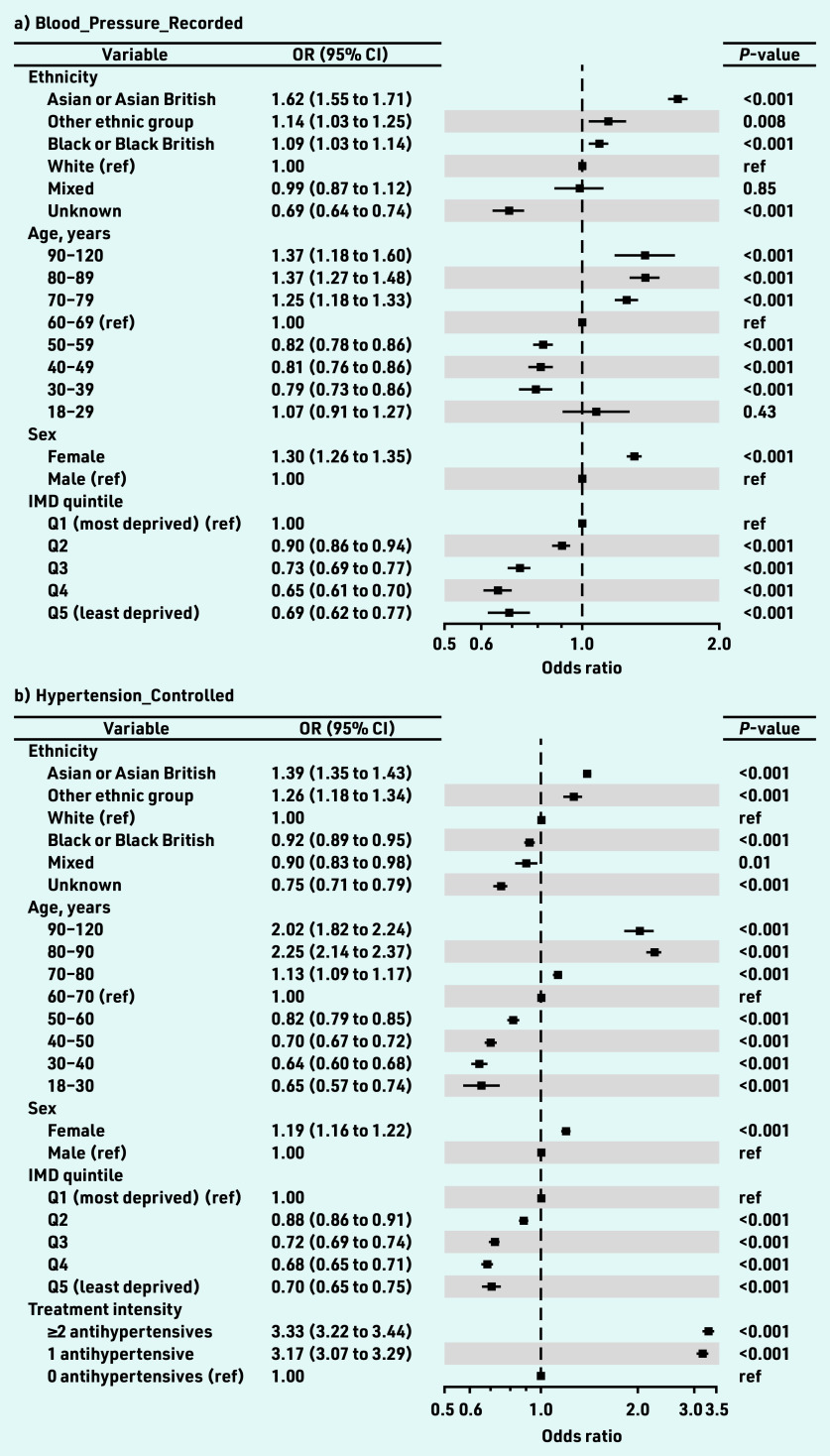

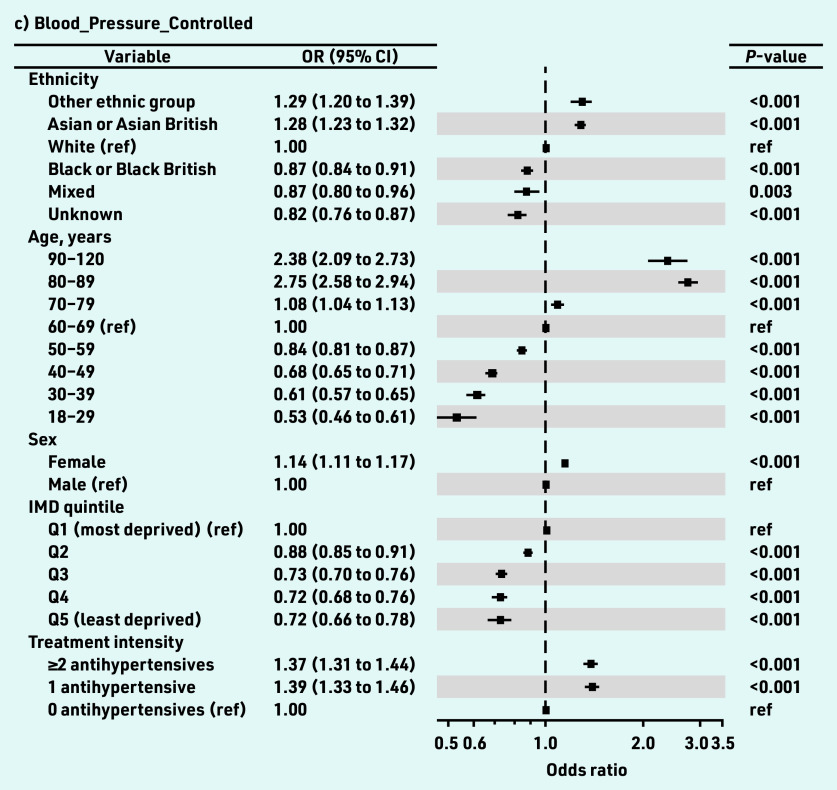
*Forest plots of ORs for a) Blood_Pressure_ Recorded; b) Hypertension_Controlled; and c) Blood_ Pressure_Controlled. The squares plot the OR relative to the reference category and the whisker bars are the 95% CI for the OR. Squares to the left of the vertical line (OR = 1.0) indicate categories in which individuals are less likely to have a blood pressure recorded in the 12 months up to and including the index date (a), or controlled blood pressure (b and c). Squares on the right indicate individuals more likely to have a recorded blood pressure (a), or a controlled blood pressure (b and c). Hypertension_Controlled (b) considers the whole cohort whereas Blood_Pressure_Controlled (c) considers only cohort members with a valid blood pressure recorded in the 12 months up to and including the index date. The ORs for treatment intensity for Blood_Pressure_Recorded are not plotted as they are significantly larger than the other ORs and affect graph clarity (1 medication: 7.83, 95% CI = 7.48 to 8.21; ≥2 medications: 9.99, 95% CI = 9.55 to 10.45; both P<0.001). OR = odds ratio. ref = reference.*

In the multivariate model, the ORs for blood pressure recording were essentially unchanged. For the Asian ethnicity group the OR was 1.62 (95% CI = 1.55 to 1.71, *P*<0.001), and for the Black ethnicity group the OR was 1.09 (95% CI = 1.03 to 1.14, *P*<0.001), as shown in Figure 2a.

### Control of hypertension and ethnicity

Hypertension_Controlled (that is, where individuals with no blood pressure recorded in the 12 months up to and including the index date were categorised as having uncontrolled blood pressure) and ethnicity were likewise modelled using univariate and multivariate models (Table 2). In the univariate model, the Asian ethnicity group was 26% more likely to have controlled hypertension than the White ethnicity group (OR 1.26, 95% CI = 1.22 to 1.30, *P*<0.001) and the Black ethnicity group was 10% less likely (OR 0.90, 95% CI = 0.87 to 0.93, *P*<0.001) to have controlled hypertension than the White ethnicity group (Table 2 and Supplementary Figure S1b).

In the multivariate model (Table 2 and Figure 2b), the Asian ethnicity group was 39% more likely to have controlled hypertension than the White ethnicity group (OR 1.39, 95% = CI 1.35 to 1.43, *P*<0.001) whereas the Black ethnicity group was 8% less likely (OR 0.92, 95% CI = 0.89 to 0.95, *P*<0.001).

### Control of blood pressure and ethnicity

The relationship between Blood_Pressure_ Controlled (that is, where only individuals with a blood pressure recorded in the 12 months up to and including the index date were considered) and ethnicity was modelled as for the two previous indicators (Table 2). In the univariate model, the Asian ethnic group was 23% more likely to have controlled blood pressure than the White ethnic group (OR 1.23, 95% CI = 1.20 to 1.27, *P*<0.001) whereas the Black ethnicity group was 8% less likely (OR 0.92, 95% CI = 0.89 to 0.95, *P*<0.001) as shown in Table 2 and Supplementary Figure S1b.

These differences were accentuated in the multivariate model (Table 2 and Figure 2c). The Asian ethnicity group was 28% more likely to have controlled blood pressure than the White ethnicity group (OR 1.28, 95% CI = 1.23 to 1.32, *P*<0.001); the Black ethnicity group was 13% less likely to have a controlled blood pressure (OR 0.87, 95% CI = 0.84 to 0.91, *P*<0.001).

### Age, IMD, sex, and treatment intensity

Data from the multivariate analysis of the Blood_Pressure_Controlled indicator (the authors’ favoured indicator of control as explained in the Method and Discussion) were considered to understand the impact of age, deprivation, sex, and treatment intensity on effective management of hypertension (Figure 2c). Compared with individuals in the (most populated) 60–69 years age bracket (25.5%, *n* = 39 854/156 296), individuals in the 30–39 years age bracket (3.9%, *n* = 6063/156 296) were 39% less likely to have controlled blood pressure (OR 0.61, 95% CI = 0.57 to 0.65, *P*<0.001). Conversely, individuals in the 80–89 years bracket (11.7%, *n* = 18 264/156 296) were 175% more likely to have controlled blood pressure (OR 2.75, 95% CI = 2.58 to 2.94, *P*<0.001). When re-grouping the data, individuals aged <50 years were 40% less likely to have controlled blood pressure than those ≥50 years (OR 0.60, 95% CI = 0.60 to 0.61, *P*≤0.001) (data not shown).

The likelihood of blood pressure control decreased with decreasing deprivation (Figure 2c). Patients in the least deprived IMD quintiles (Q4 and Q5) were 28% less likely than patients in the most deprived quintile (Q1) to have controlled blood pressure (ORs 0.72, 95% CI = 0.68 to 0.76 and 0.72, 95% CI = 0.66 to 0.78, respectively, both *P*<0.001). Females were 14% more likely to have controlled blood pressure than males (Figure 2c; OR 1.14. 95% CI = 1.11 to 1.17, *P*<0.001).

Relative to untreated individuals, the likelihood of blood pressure control increased with treatment intensity by approximately 38% regardless of treatment intensity (Figure 2c; ORs: 1 antihypertensive 1.39, 95% CI = 1.33 to 1.46; ≥2 antihypertensives 1.37, 95% CI = 1.31 to 1.44, both *P*<0.001).

#### Ethnicity and treatment intensity

The distribution of treatment intensities by ethnicity group in those with controlled versus uncontrolled blood pressures (that is, above target, unrecorded, or invalid blood pressure recordings) is shown in Supplementary Figure S2.

Individuals in the Asian ethnicity group were the least likely to be untreated in both the uncontrolled and controlled blood pressure groups: 11.9% (*n* = 908/7639) and 7.3% (*n* = 2627/36 169), respectively. Individuals in the Black ethnicity group were similarly or less likely to be untreated or be on a single antihypertensive medication than the White ethnicity group. In the controlled blood pressure group, 8.5% (*n* = 2016/23 850) of the Black ethnicity group were untreated and 32.9% (*n* = 7851/23 850) on a single agent (compared with 9.4% [*n* = 4084/43 659] and 34.4% [*n* = 15 033/43 659] for the White ethnicity group). In the uncontrolled blood pressure group, 13.6% (*n* = 918/6731) of the Black ethnicity group were untreated and 31.7% (*n* = 2133/6731) on a single agent compared with 13.0% (*n* = 1329/10 233) and 36.0% (*n* = 3685/10 233) for the White ethnicity group.

**Figure 3.**
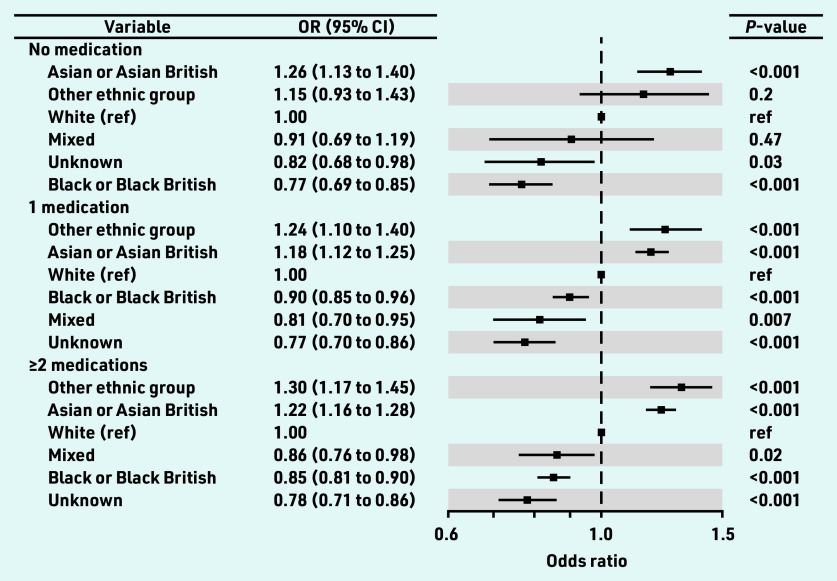
*ORs (Blood_Pressure_Controlled) by ethnicity for patients on 0, 1, and ≥2 antihypertensive medications. OR = odds ratio. ref = reference.*

A multivariate analysis was conducted for the Blood_Pressure_Controlled indicator for: a) untreated individuals; b) individuals on one class of antihypertensive; and c) individuals on ≥2 classes of antihypertensives. The ORs by ethnicity group for this treatment intensity subanalysis are shown in Figure 3. Relative to the White ethnicity group, and regardless of the number of medications they were on, individuals in the Asian ethnicity group were 12%–26% more likely to have controlled blood pressure: 0 medications: OR 1.26, 95% CI = 1.13 to 1.40, *P*<0.001; 1 medication: OR 1.18, 95% CI = 1.12 to 1.25, *P*<0.001; and ≥2 medications: OR 1.22, 95% CI = 1.16 to 1.28, *P*<0.001. The Black ethnicity group was always less likely (10%–23%) to have controlled blood pressure (0 medications: OR 0.77, 95% CI = 0.69 to 0.85, *P*<0.001; 1 medication: OR 0.90, 95% CI = 0.85 to 0.96, *P*<0.001; ≥2 medications: OR 0.85, 95% CI = 0.81 to 0.90, *P*<0.001).

## DISCUSSION

### Summary

This cross-sectional study of an unselected, large cohort of 156 296 adult patients with hypertension from 190 GP practices in North East London, identified inequities in the monitoring and control of hypertension by ethnicity, age, sex, and deprivation.

The Black ethnicity group was more likely to have uncontrolled hypertension than White or Asian ethnicity groups. This was not because of less frequent recording of blood pressure in the Black ethnicity group, as these individuals were in fact more likely to have a blood pressure recorded within 1 year than those in the White ethnicity group — nor was this finding owing to associations with age, sex, or deprivation. Regardless of treatment intensity, control of blood pressure in the Black ethnicity group was worse, and in the Asian ethnicity group better, than in the White ethnicity group. It is of note that despite better control of hypertension in the Asian ethnicity group, CVD mortality is higher in this group than in both the White and Black ethnicity groups.^20^

These data suggest that lower levels of blood pressure control in the Black ethnicity group cannot be directly attributed to relative underrecording of blood pressure or less intensive treatment. Other possible factors include unidentified confounding factors, differences in age at diagnosis, initial treatment choice and dosage, treatment escalation, adherence, and physiological treatment resistance.^10^^,^^21^^–^23 Issues relating to medication cost burden on working-age adults and adherence may also be contributory factors in particular in younger people (aged <50 years).^10^^,^^24^^,^^25^

Patients with hypertension aged <50 years were 40% less likely than those older to have controlled hypertension. Given that 16.8% of the cohort was aged <50 years (with more potential years at risk), this represents a notable treatment inequity.^24^ Similar concerns have been expressed about statin undertreatment in younger patients.^26^

Lastly, this study highlights the importance of indicator choice in assessing cohort-level management of hypertension. The difference between Blood_Pressure_Controlled and the QOF derived Hypertension_Controlled indicators has been previously noted as reflecting missing recordings rather than reduction in control.^7^ The QOF HYP003 and HYP007 indicators may be suitable for performance assessment in incentive programmes but do not provide reliable indicators of the management of hypertension — considering both Blood_Pressure_Recorded (an indicator of monitoring) and Blood_ Pressure_Controlled (an assumption-free indicator of control) is preferable.

### Strengths and limitations

The study cohort was unselected, included almost all (95%) general practices in the region, and had a high level (96%) of recorded ethnicity in an ethnically diverse population with broad representation of the three principal ethnic groups discussed. However, it is important to be mindful that high-level ethnicity groupings can conceal underlying heterogeneity between constituent ethnicity groups.^27^ This is relevant because subgroups of, for example, South Asian, includes Indian, Bangladeshi, and Pakistani, and it is possible that one of these groups could have higher or lower BP than another; similarly, Black ethnic group might conceal differences in a Black subgroup such as Kalahari bushmen.

The study was cross-sectional and trajectories of the patient groups through time were not addressed. Treatment changes within 6 months would not be captured accurately, possibly overestimating treatment intensity in a small number of patients. In North East London, 72.6% of the population were in the two most deprived quintiles, and only 10.6% in the two most affluent. The urban density and the fact that the wealthy and deprived often live in close proximity means that area-level measures such as IMD quintile reduce gradients between extremes of deprivation. In addition, the most affluent may be away from their London residences for prolonged periods or access alternative health services that may further influence blood pressure recording and control, and contribute to apparent poor management in this group.

### Comparison with existing literature

The current study confirms previous findings of inequities in the control of blood pressure in patients with hypertension, with poorer control in the Black ethnicity group and better control in the Asian ethnicity group in comparison with the White ethnicity group, as well as better control in females compared with males.^8^^–^12 This study found younger individuals had poorer control than older individuals, a finding not replicated in a cross-sectional population- based study of almost 100 000 individuals with hypertension enrolled in the UK Biobank database (although that study did not consider individuals aged <40 years).^9^ In the current study, the Black ethnicity group had similar access to primary care-based blood pressure recording as other ethnicity groups, a finding similar to that in a South East London-based study.^8^

In a UK-wide study by Eastwood *et al,* comparing management of people with newly diagnosed hypertension from European, South Asian, and African/African Caribbean ethnicities, the findings of poorer control in the Black ethnicity group was similar to the current study, but the South Asian group was found to have similar levels of blood pressure monitoring and control in comparison with other ethnic groups.^10^ The current study found higher levels of blood pressure monitoring and of blood pressure control in the Asian ethnicity group than in the White ethnicity group. However, the UK-wide study considered only recently diagnosed patients, which may account for differences in findings in the South Asian ethnicity group.

The current study showed that, regardless of treatment intensity, the Black ethnicity group was less likely to have controlled hypertension. When considering only individuals who were adherent to their antihypertensive treatment, Eastwood *et al*^10^ did not find differences between blood pressure control in the African/African Caribbean and European groups. However, cost of prescriptions are of concern as a possible contributing factor in working age adults.^28^

Two UK-based studies investigated the impact of ethnicity on medication adherence, although neither considered antihypertensive medication. The first considered diabetes, dyslipidaemia, or hypothyroidism medication within an ethnically diverse inner-city cohort and found lower adherence in Asian or Black patients.^29^ The second considered persistence with oral medication for type 2 diabetes. Relative to the White ethnicity group, non-persistence was more likely in the Black and the Asian ethnic groups (hazard ratios 1.83 and 1.53, respectively).^30^ Such findings do not explain the higher likelihood of controlled blood pressure in the current study’s Asian ethnicity group. Furthermore, this study found no evidence that the Black ethnicity group was undertreated.

The age-standardised mortality rates (ASMR) from ischaemic heart disease (IHD) for the Bangladeshi, Pakistani, and Indian ethnicity groups are significantly higher than in the White ethnicity group — who in turn have a higher ASMR than the Black ethnicity group.^20^ Given the higher risk of cardiac death, there should be no complacency in the need to improve blood pressure control in the Asian ethnicity group and the higher levels of stroke in the Black ethnicity group raise similar concerns.

### Implications for research and practice

Further research is needed to understand ethnic differences in IHD and stroke mortality, and the relevance of thresholds for blood pressure control or ethnicity- specific treatment pathways.^22^^,^^27^^,^^31^

In North East London and the UK more generally, a focus on the optimisation of blood pressure control in people of Black ethnicity and in younger people could be an important step in addressing the monitor and control of hypertension inequities.

The impact of medication costs in younger working age groups deserves further attention and, in particular, the role of single pill combination therapies that reduce patient costs and improve adherence.

Easier access to monitoring of blood pressure is relevant to all people with hypertension particularly those of working age.^4^^,^^32^^,^^33^

## Data

The data are not publicly available. The full results are presented in the Supplementary Tables.
